# The SARS-CoV-2 Nucleoprotein Induces Innate Memory in Human Monocytes

**DOI:** 10.3389/fimmu.2022.963627

**Published:** 2022-07-19

**Authors:** Patricia Urbán, Paola Italiani, Diana Boraschi, Sabrina Gioria

**Affiliations:** ^1^ European Commission, Joint Research Centre (JRC), Ispra, Italy; ^2^ Institute of Protein Biochemistry and Cell Biology (IBBC), National Research Council (CNR), Napoli, Italy; ^3^ Stazione Zoologica Anton Dohrn, Napoli, Italy; ^4^ Shenzhen Institute of Advanced Technologies (SIAT), Chinese Academy of Sciences (CAS), Shenzhen, China

**Keywords:** SARS-CoV-2, nucleoprotein, innate immunity, innate memory, monocytes, cytokines

## Abstract

The interaction of SARS-CoV-2 with the human immune system is at the basis of the positive or negative outcome of the infection. Monocytes and macrophages, which are major innate immune/inflammatory effector cells, are not directly infected by SARS-CoV-2, however they can react to the virus and mount a strong reaction. Whether this first interaction and reaction may bias innate reactivity to re-challenge, a phenomenon known as innate memory, is currently unexplored and may be part of the long-term sequelae of COVID-19. Here, we have tested the capacity of SARS-CoV-2 and some of its proteins to induce innate memory in human monocytes *in vitro*. Our preliminary results show that the Spike protein subunits S1 and S2 and the entire heat-inactivated virus have no substantial effect. Conversely, monocytes pre-exposed to the nucleocapsid N protein react to subsequent viral or bacterial challenges with an increased production of anti-inflammatory IL-1Ra, a response profile suggesting a milder response to new infections.

## Introduction

The novel coronavirus SARS-CoV-2, which suddenly emerged in December 2019, is still haunting mankind and has affected not only the healthcare systems but also the global socio-economic balances (1–3). COVID-19, the disease caused by the virus, was designated as a global pandemic by the World Health Organization, with more than 534 million confirmed cases and over 6.3 million confirmed deaths, as of June 2022 (4). Taking advantage of the recent progress in virology, molecular biology and pharmacology, and thanks to an amazing effort of the international scientific community, both in academia and industry, and a huge resource allocation, we were rapidly able to dissect and understand the SARS-CoV-2 structure, functions, lifecycle, and pathological characteristics (5–9). This led in a very short time to vaccine development and to several pharmacological approaches to treat or reduce the severity of patients’ symptoms (2, 10, 11).

Despite the rapid and huge progress in understanding the interactions between the virus and the human immune system, much is still unknown to explain/predict the variability of immune responses that determines different susceptibility to severe effects, reactivity to re-infection, or response to vaccination.

Specific antiviral immunity is mainly based on the development of neutralizing antibodies and cytotoxic CD8+ T cells, while innate immune reactions encompass the activation of inflammatory protective responses triggered by recognition of viral patterns by membrane and cytoplasmic pattern-recognition receptors (12). While vaccination strategies are currently based on the generation of specific anti-viral immune memory, much less emphasis has been dedicated to harnessing the protective potential of innate immunity and its memory capacity.

The concept of innate immune memory, i.e., a change in the non-specific reactivity to challenges of innate immune cells previously exposed to various stimuli, is well known in plants, invertebrates and also in vertebrates (13–15). Thus, it can be hypothesized that innate memory, induced by previous exposure to infections or other challenges (including vaccination), may participate to the effectiveness of subsequent defensive innate responses in a personalized fashion, which is dependent on individual history of pathogen/antigen exposure: the “immunobiography” (16).

While abundant information is available on the specific functions and kinetics of the adaptive immune response to SARS-CoV-2 (17–19), less is established regarding the balance between protective and detrimental effects of innate immune responses in COVID-19. A general increase in the circulating levels of inflammatory cytokines has been observed (possibly secondary to barrier disruption and release of bacterial products) in parallel to downregulation of myeloid cells’ markers and function (20). There is clear evidence that the variability in innate immune system components is a main contributor to the heterogeneous disease course observed for COVID-19 (20–22) and in the response to vaccination (23). Thus, it is fundamental to understand if and how interaction of innate cells with SARS-CoV-2 induces an innate memory, and whether such memory may contribute to future protection or to post-infection pathologies, as suggested by epigenetic studies (24).

Here, we provide preliminary evidence that the SARS-CoV-2 nucleocapsid protein N, as opposed to other viral components, can induce innate memory in human primary monocytes in culture. This memory is limited to a significant increase in the production of the anti-inflammatory cytokine IL-1Ra in response challenges, suggesting a less inflammatory secondary reactivity. On this basis, we hypothesize that innate memory to viral components may contribute to the overall response to subsequent challenges (viral or bacterial infections or re-infections), including response to vaccination. The large inter-individual variability suggests the needs for a personalized assessment, in order to predict the features of innate/inflammatory reactivity to future challenges in previously infected individuals.

## Materials and Methods

### Selection of Stimuli

The human recombinant coronavirus SARS-CoV-2 nucleocapsid protein (N; ab272107) was expressed in *E. coli* with a C-terminal His tag. Expression in *E. coli* produces a non-glycosylated protein, similar to the “natural” protein. Human recombinant Spike glyco-protein subunit 1 (S1; ab 272105) and subunit 2 (S2; ab272106) were expressed in HEK 293 cells as chimeras with a C-terminal Fc tag. Recombinant proteins were purchased from Abcam (Milan, Italy). LPS contamination was checked in-house with the chromo-genic LAL assay Pyros Kinetix^®^ Flex (Associates of Cape Cod, Inc., East Falmouth, MA, USA). The endotoxin contamination was 353 EU/mg for N, 26 EU/mg for S1 and 7 EU/mg for S2. Heat-inactivated SARS-CoV-2 (ATCC VR-1986HK) was obtained from LGC standards (Milan, Italy); LPS from E. coli O55:B5 was from Sigma Aldrich^®^ (Merck KGaA, St. Louis, MO, USA); the TLR7/8 agonist Resiquimod (R848; cat. tlrl-rR848, purity ≥ 95% by UHPLC) was purchased from InvivoGen (San Diego, CA, USA). R848 was devoid of TLR2 (lipoproteins) and TLR4 agonist activity, tested on HEK-Blue TLR2 and TLR4 cells.

Concentration of viral stimuli to be used in culture was based on preliminary dose-response experiments, viability assessment and LAL results, while concentrations of reference stimuli (LPS and R848) were selected based on previous experience and *ad hoc* dose-response assessment (data not shown). Thus, the same concentration of 1 µg/mL was selected for N, S1 and S2, which corresponded to an endotoxin contamination of 0.35, 0.03 and 0.01 EU/mL. Since 1 EU roughly corresponds to 100 pg of LPS, the LPS contamination of N was estimated around 35 pg/mL in the assay, a concentration unable to induce monocyte activation in our hands. For the whole heat-inactivated virus, 5x10^5^ RNA genome copies were used as stimulus in culture, based on preliminary dose-response experiments (data not shown).

### Human Monocyte Isolation

Blood was obtained from anonymized healthy SARS-CoV-2 negative non-vaccinated donors, upon informed consent and in agreement with the Declaration of Helsinki. The protocol was approved by the Regional Ethics Committee for Clinical Experimentation of the Tuscany Region (Ethics Committee Register n. 14,914 of May 16, 2019). Monocytes were isolated by CD14 positive selection with magnetic microbeads (Miltenyi Biotec, Bergisch Gladbach, Germany) from peripheral blood mononuclear cells (PBMC), obtained by Ficoll-Paque gradient density separation (GE Healthcare, Bio-Sciences AB, Uppsala, Sweden). Monocyte preparations used in the experiments were > 95% viable and > 95% pure (assessed by trypan blue exclusion and cytosmears).

Monocytes were cultured in culture medium (RPMI 1640 + Glutamax-I; GIBCO by Life Technologies, Paisley, UK) supplemented with 50 µg/mL gentamicin sulfate (GIBCO) and 5% heat-inactivated human AB serum (Sigma-Aldrich). Cells (1x10^5^) were seeded in a final volume of 100 µL in 96-wells flat bottom plates (Corning^®^ Costar^®^; Corning Inc. Life Sciences, Oneonta, NY, USA) at 37°C in moist air with 5% CO_2_. Monocyte stimulation was performed after overnight resting.

### Human Monocyte Activation and Induction of Innate Memory

For assessing the primary response to stimulation, monocytes were exposed for 24 h to culture medium alone (medium/negative control) or containing LPS (positive bacterial control, 1 ng/mL), R848 (positive viral control, 0.5 µg/mL), heat-inactivated SARS-CoV-2 virus (5x10^5^ RNA copies), N (1 µg/mL), S1 (1 µg/mL) or S2 (1 µg/mL). Cell viability, measured both as lack of LDH release (LDH-Cytotoxicity Colorimetric Assay Kit; BioVision, Inc., Milpitas, CA, USA) and as metabolic activity (reduction of MTT to formazan) (25) was unaffected by treatment (data not shown). At the end of the exposure time, all supernatants were collected. For memory experiments, cells were then washed and cultured with fresh culture medium for 7 additional days (one medium change after 4 days). During this period, after the activation induced by previous stimulation subsides, cells return to their baseline status (as determined by evaluation of inflammation-related cytokines in the supernatant; data not shown). At the end of the resting phase, the supernatant was collected and cells were challenged for 24 h with fresh medium alone or containing 5 ng/mL LPS, 2.5 µg/mL R848, or 5 µg/mL N (i.e., a 5x higher concentration than in the primary stimulation), according to the standard protocols for *in vitro* innate memory assessment (14, 15). All supernatants (after the first stimulation, after the resting phase and after the challenge phase) were frozen at -80°C for subsequent cytokine analysis. By visual inspection, cell viability and cell number did not substantially change in response to the different treatments.

### Cytokine Analysis by Multiplex ELISA

Samples were tested for the presence of cytokines and chemokines by commercial ELISA-based microarrays that simultaneously measure multiple proteins in a single sample aliquot. Multiplex Bio-Plex Pro™ Human Cytokine 8-plex Assay (cat. M50000007A) was used for assessing the production of IL-2, IL-4, IL-6, IL-8, IL-10, TNFα, IFN-γ, and GM-CSF. Singleplex for IL-1β (cat. 171B5001M) and IL-1Ra (cat. 171B5002M) were also included. Samples were run according to the manufacturer’s instructions. Cytokines were analyzed with the Bio-Plex200 System using the Bio-Plex Manager™ software, and data were analyzed by the Bio-Plex Data Pro™ software, using five-parametric curve fitting. For each cytokine, assay ranges and LOD were provided by the manufacturer. All reagents and instruments, including Washing Station and Shaker Incubator, were from BIO-RAD Laboratories, Inc. (Hercules, CA, USA). Two repeated measurements were made for each marker for each donor. The symbols reported in the figures are the averages of such repeated measurements.

### Statistical Analysis

Data were analyzed using the GraphPad Prism 6.01 software (GraphPad Inc., La Jolla, CA, USA). For cytokine production, results are presented as ng produced cytokine/10^6^ plated monocytes. Results from individual donors are reported as mean values of 2-3 replicates (each tested with technical duplicates in ELISA). Average values of individual donors’ data are reported as light grey columns. Statistical significance of differences is indicated by *p* values, calculated using one way ANOVA and Dunnett’s Multiple Comparison.

## Results

### Primary Response of Human Monocytes to Inactivated SARS-CoV-2 or Its Components

**Figure 1:**
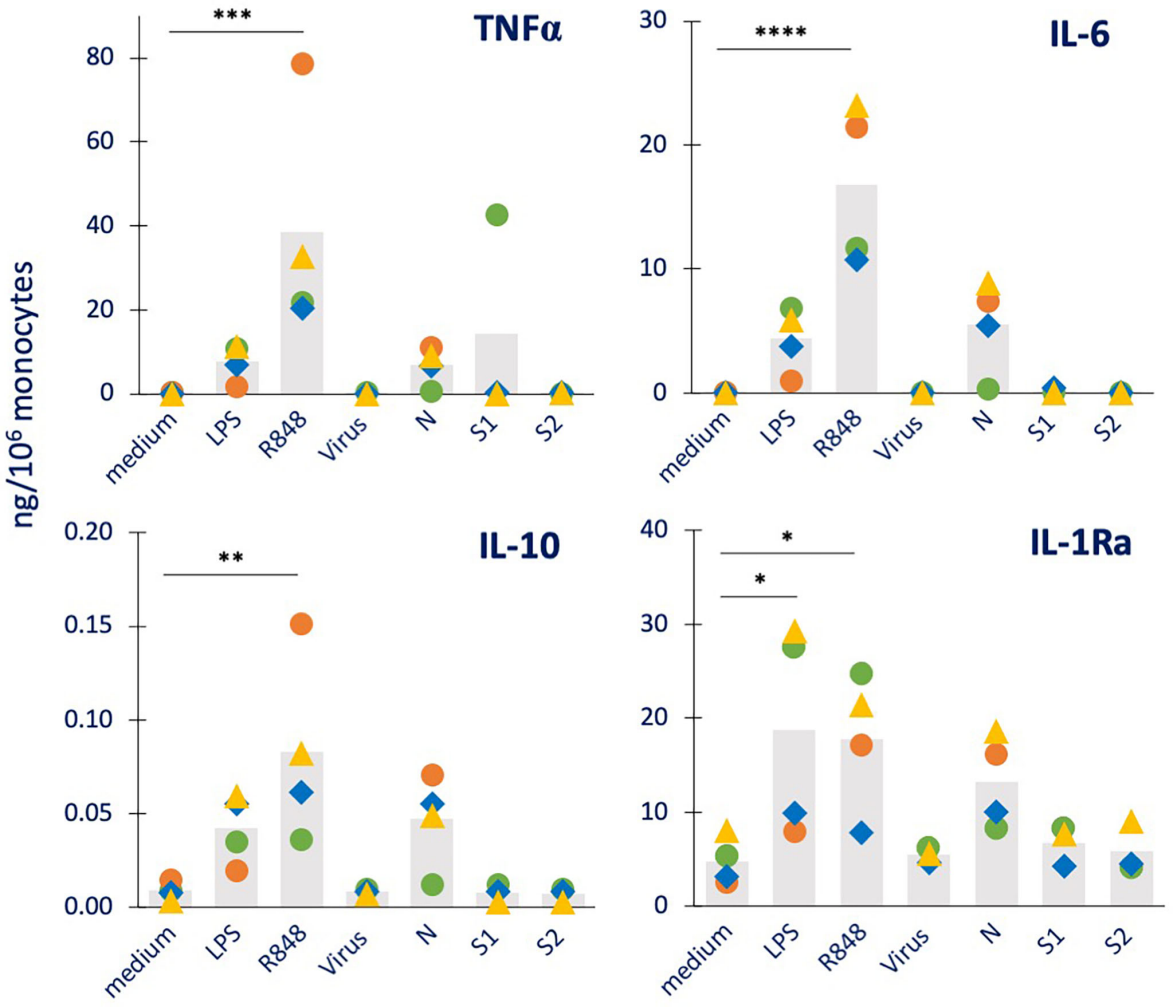
Primary innate immune primary response to inactivated SARS-CoV-2 or its proteins in human monocytes. Human monocytes isolated from blood of four individual donors (green, red, blue, and yellow symbols) were cultured for 24 h in culture medium alone or containing the inactivated SARS-CoV-2 virus (5 x10^5^ copies), or the viral proteins N, S1, S2 (all at 1 µg/mL). The production of TNFα (upper left), IL-6 (upper right), IL-10 (lower left) and IL-1Ra (lower right) was measured in the 24 h supernatants by ELISA. Medium alone was used as baseline value, LPS (1 ng/mL) and R848 (0.5 µg/mL) were used as positive controls. Data are presented as individual donors’ values (colored symbols) and as mean of the individual values (gray columns). Statistical significance: * *p <*0.05; ** *p <*0.01; *** *p <*0.01; **** *p <*0.0001.

The primary response of human monocytes to different SARS-CoV-2 stimuli was assessed after exposure *in vitro* for 24 h. Monocytes are key innate immune cells responsible of inflammatory defensive responses, and their activation was evaluated in terms of production of four innate cytokines, the inflammatory factors TNFα and IL-6, and the anti-inflammatory cytokines IL-10 and IL-1Ra (**Figure 1**). As positive control, cells were exposed to LPS or R848, potent activators of human monocyte innate/inflammatory responses that mimic bacterial and viral challenges, respectively. The concentrations of LPS (1 ng/mL) and R848 (0.5 µg/mL) were selected in order to induce a measurable but not maximal response (data not shown). The viral agents used were the heat-inactivated SARS-CoV-2 virus (5x10^5^ RNA genomic copies/well; 5:1 vs. monocytes), the nucleocapsid protein N (1 µg/mL), the S1 subunit of the Spike protein (responsible for viral binding to target cells; 1 µg/mL) and the S2 subunit of the Spike protein (responsible for viral entry in target cells; 1 µg/mL). The endotoxin contamination of the recombinant viral proteins was below monocyte activation threshold (see Materials and Methods). Monocytes from four SARS-CoV-2 negative non-vaccinated donors were tested.

As shown in **Figure 1**, both LPS and R848 were able to induce a substantial production of the inflammatory factors TNFα and IL-6 and of the anti-inflammatory cytokine IL-10, although with a high inter-individual variability (which in some cases did not allow for reaching statistical significance). For the other anti-inflammatory cytokine IL-1Ra, as expected, a measurable constitutive production was evident in unstimulated cells, which was increased by exposure to LPS or R848. Among the viral agents, only N showed the capacity to stimulate monocytes (in 2/3 of 4 donors), whereas the inactivated whole virus and the two Spike proteins were essentially inactive.

The production by monocytes of other inflammation-related factors was also examined. Data for the inflammatory cytokine IL-1β, the chemokine IL-8, the immune interferon IFN-γ and the growth factor GM-CSF are reported in **Supplementary Figure S1**, while the production of the T cell cytokines IL-2 and IL-4 was undetectable (data not shown). Again, N was the only viral factor able to activate monocyte responses (evident for IL-8 and GM-CSF production in 3 out of 4 donors), which were in the same range as those induced by the positive controls LPS and R848. These results partially confirm a previous report, showing that N could induce the production of IL-6 and IL-10 in human monocytes, but not TNFα and IL-1β (26). At variance with the same study, here we could not see any effect by S1. The differences in the recombinant protein constructs and in the exposure time (1 *vs*. 3-5 days), and the fact that the endotoxin contamination was not assessed in the other study may explain the different result.

### Secondary Memory Response of Human Monocytes Primed With Inactivated SARS-CoV-2 or Its Components

**Figure 2:**
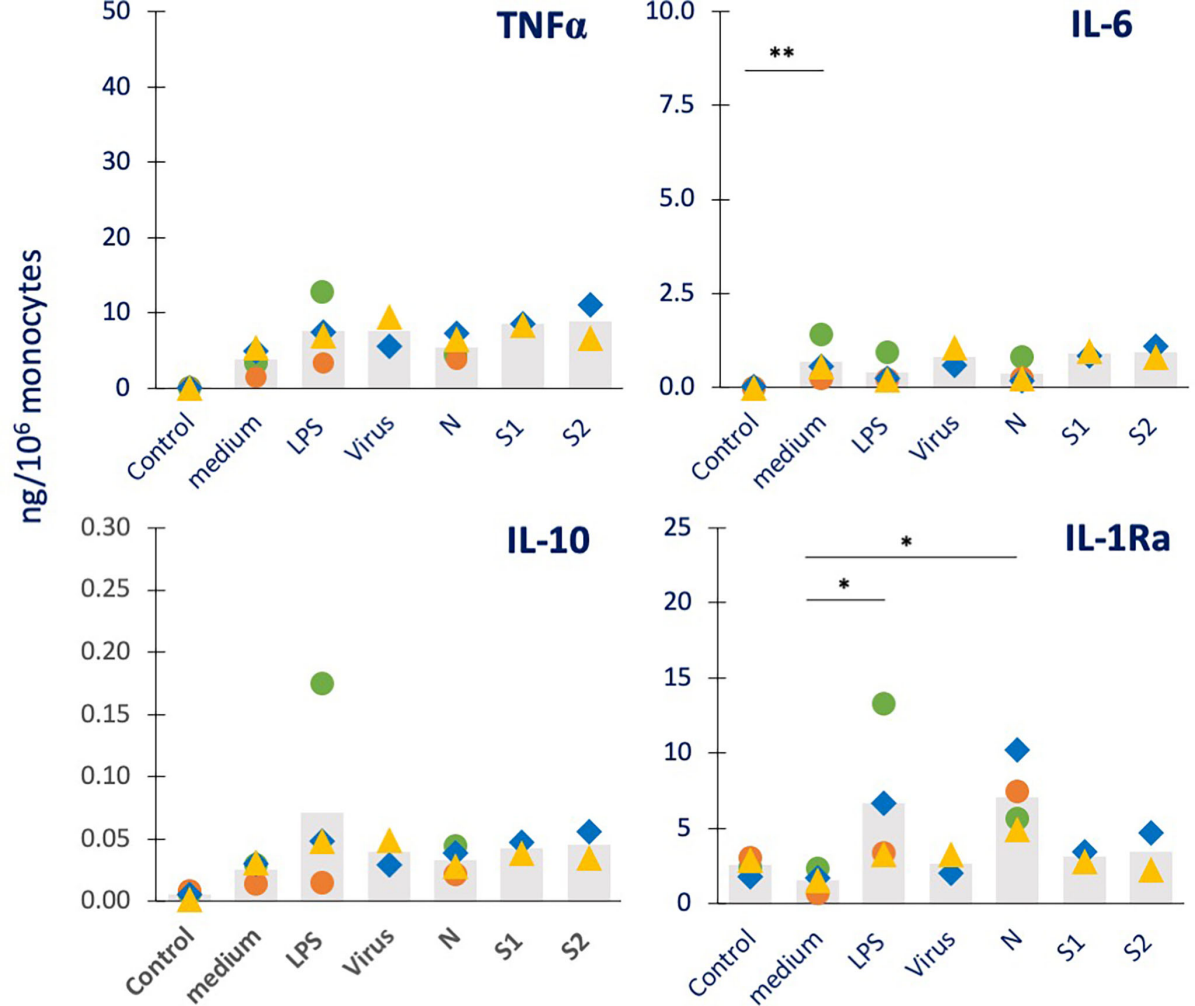
Innate immune memory response to a bacterial challenge in human monocytes primed with inactivated SARS-CoV-2 or its proteins. Human monocytes isolated from blood of four individual donors (green, red, blue, and yellow symbols) were cultured for 24 h in culture medium alone (column “medium”) or containing LPS (1 ng/mL, positive bacterial control, column “LPS”), the inactivated SARS-CoV-2 virus (5 x10^5^ copies, column “virus”), N, S1, or S2 (all at 1 µg/mL; columns “N”, “S1” and “S2”). Cells were then washed and rested for 7 days in the absence of stimuli, then challenged for 24 h in fresh medium alone (column “control”) or containing 5 ng/mL LPS. The column “control” values from cells that received no challenge are included in each panel as “control” and encompasses the values from primed and unprimed cells that received no challenge, i.e., fresh medium alone. These values did not differ between primed and unprimed cells, confirming the return to baseline after the resting period. The production of TNFα (upper left), IL-6 (upper right), IL-10 (lower left) and IL-1Ra (lower right) was measured in the 24 h supernatants by ELISA. Data are presented as individual donors’ values (colored symbols) and as mean of the individual values (grey columns). Statistical significance: * *p <*0.05; ** *p <*0.01.

**Figure 3:**
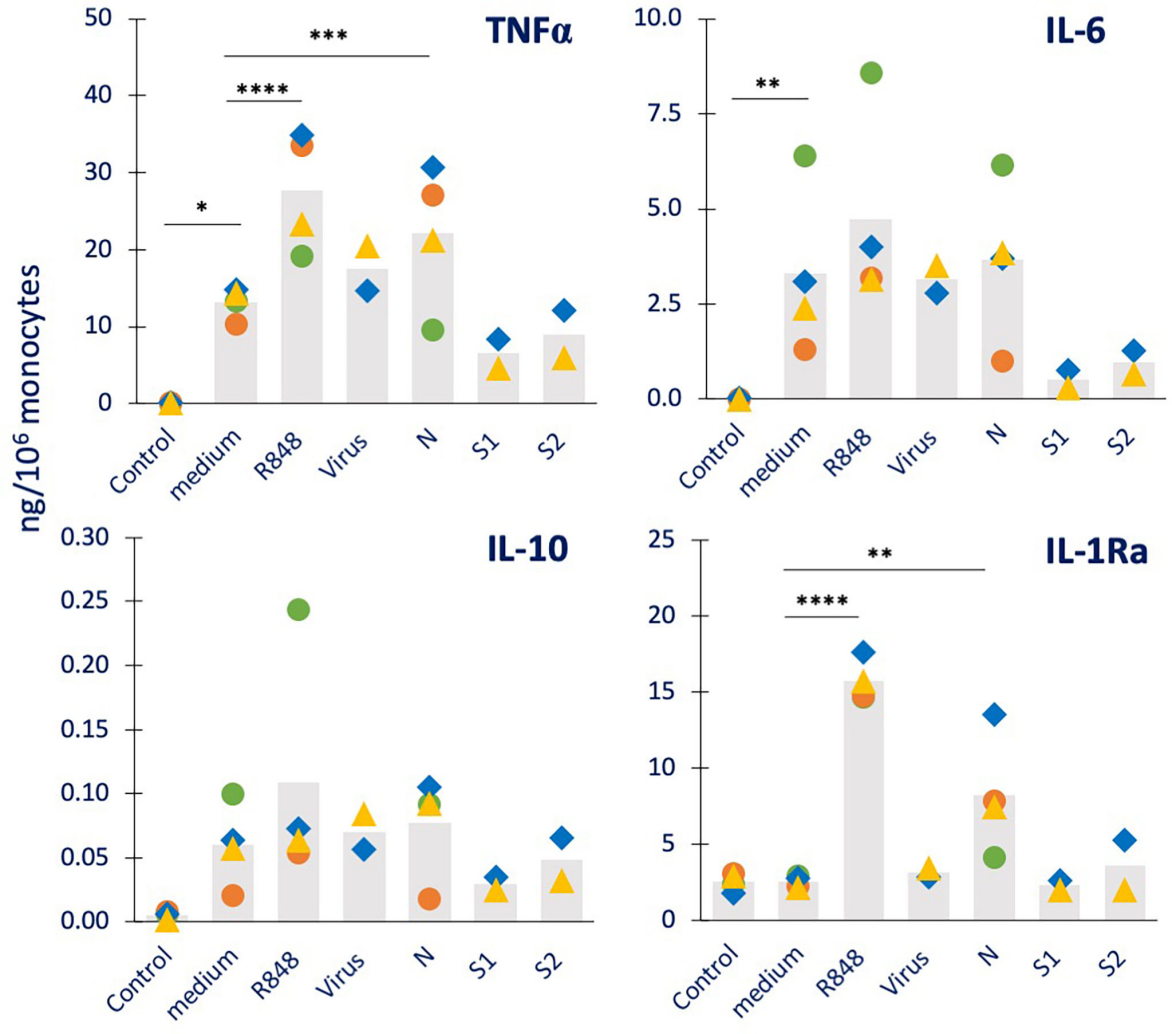
Innate immune memory response to a viral challenge in human monocytes primed with inactivated SARS-CoV-2 or its proteins. Human monocytes isolated from blood of four individual donors (green, red, blue, and yellow symbols) were cultured for 24 h in culture medium alone or containing R848 (0.5 µg/mL, positive viral control), the inactivated SARS-CoV-2 virus (5 x10^5^ copies), or the viral proteins N, S1, and S2 (all at 1 µg/mL). Cells were then washed and rested for 7 days in the absence of stimuli, then challenged for 24 h in fresh medium alone or containing 2.5 µg/mL R848. The production of TNFα (upper left), IL-6 (upper right), IL-10 (lower left) and IL-1Ra (lower right) was measured in the 24 h supernatants by ELISA. The values from cells that received no challenge are included in each panel as “control” and encompass the values obtained from primed and unprimed cells (which did not differ, confirming the return to baseline after the resting period). Data are presented as individual donors’ values (colored symbols) and as mean of the individual values (gray columns). Statistical significance: * *p <*0.05; ** *p <*0.01; *** *p* < 0.001; **** *p <*0.0001.

After the primary response (**Figures 1** and **S1**), cells were washed and cultured for 7 additional days in fresh culture medium to allow extinction of activation and return to baseline. The culture medium was refreshed after 4 days. The extinction of cell activation was confirmed by examining the production of cytokines released in the culture medium at the end of the resting period (representing the cytokine release in the last 3 days of resting) (data not shown). After the extinction period, cells were either exposed to medium alone (control) or challenged with 5x higher concentration of LPS (representing a bacterial challenge) or R848 (representing a viral challenge), in order to assess the development of a memory response able to react to more severe challenges. LPS priming was used as control of LPS challenge, while R848 priming was used as control of R848 challenge. As for the primary response, the memory response was assessed in terms of production of inflammatory and anti-inflammatory cytokines, and the results are reported in **Figures 2** and **3** for the major inflammatory (TNFα, IL-6) and anti-inflammatory (IL-10, IL-1Ra) cytokines.

As expected, LPS challenge of medium-primed cells showed a general induction of TNFα, IL-6 and IL-10 production, but no increase over the substantial baseline production of IL-1Ra (columns “medium” vs. “Control” in **Figure 2**). Although the increase did not always reach statistical significance on average, this was evident at the individual donor’s level. LPS-primed cells did not show the development of a tolerance memory response (columns “LPS” *vs.* “medium”) relative to the inflammatory cytokine TNFα, while this was small but detectable in 2/4 donors for IL-6, confirming the variability in the development of LPS tolerance already observed in other subjects (15, 27, 28). A tendency to a potentiated response could be observed in LPS-primed cells in terms of production of the anti-inflammatory factor IL-10 (although not reaching statistical significance), while a significant increase in IL-1Ra was evident. Priming with the inactivated virus did not have substantial effects on the response to the LPS challenge (columns “Virus” *vs.* “medium”). When examining the memory-inducing capacity of viral proteins, it was observed that the S1 and S2 subunits of the Spike protein had no memory-inducing activity (columns “S1” and “S2” *vs.* “medium”), similar to the inactivated virus. Priming with the nucleocapsid protein N, on the other hand, induced a significant and considerable potentiation of IL-1Ra production (columns “N” *vs.* “medium”). When examining the memory effects of virus or viral components on other innate immune factors induced by the bacterial challenge LPS (**Supplementary Figure S2**), we observed that neither LPS nor virus/viral components had clear effects on the production of the inflammatory cytokine IL-1β, the chemokine IL-8, the immune interferon IFN-γ and the growth factor GM-CSF, again with inter-individual variability of response.

We also assessed the capacity of SARS-CoV-2 and its components to induce innate memory to a viral challenge, using R848 as prototypical viral agent (**Figure 3**). As a control, priming with R848 was included. The results show that unprimed monocytes respond to R848 challenge with a potent production of TNFα, IL-6 and IL-10 (in 3/4 donors), generally higher than that induced by the bacterial challenge. Conversely, the R848 challenge was completely inactive in modulating the constitutive production of IL-1Ra (columns “medium” *vs.* “Control”). Priming with R848 potentiated the secondary response to the R848 (columns “R848” *vs.* “medium”) in all donors for TNFα, IL-6 and IL-1Ra, and in 2/4 donors for IL-10 (although the average increase reached statistical significance only for TNFα and IL-1Ra). Priming with the inactivated virus had little/no effect on the secondary response to the R848 virus-like challenge (columns “virus” *vs.* “medium”). Priming with the nucleocapsid protein N showed potentiation of the memory response to R848 in terms of IL-1Ra production (in all 4 donors), while having limited/no effect of the secondary production of TNFα, IL-6 and IL-10 (columns “N” *vs.* “medium”). The priming with the Spike protein subunits S1 and S2 showed no substantial variations relative to controls (columns “S1” and “S2” *vs.* “medium”). We have additionally examined other four cytokines (IL-1β, IL-8, IFN-γ, GM-CSF; **Supplementary Figure S3**). All four factors are produced by medium-primed cells in response to R848 (columns “medium” *vs.* “Control”). While the production of IL-8 was never significantly modified by priming with any agent (upper right panel), priming with R848 potentiates the production of IL-1β, IFN-γ and GM-CSF (columns “R848” *vs.* “medium” in the left and lower right panels). As in other cases, priming with the inactivated virus had no memory effect (columns “Virus” *vs.* “medium”). Priming with N showed a potentiation of the response to R848 in terms of IL-1β production in all donors and GM-CSF in 3/4 donors (columns “N” *vs.* “medium”).

**Figure 4:**
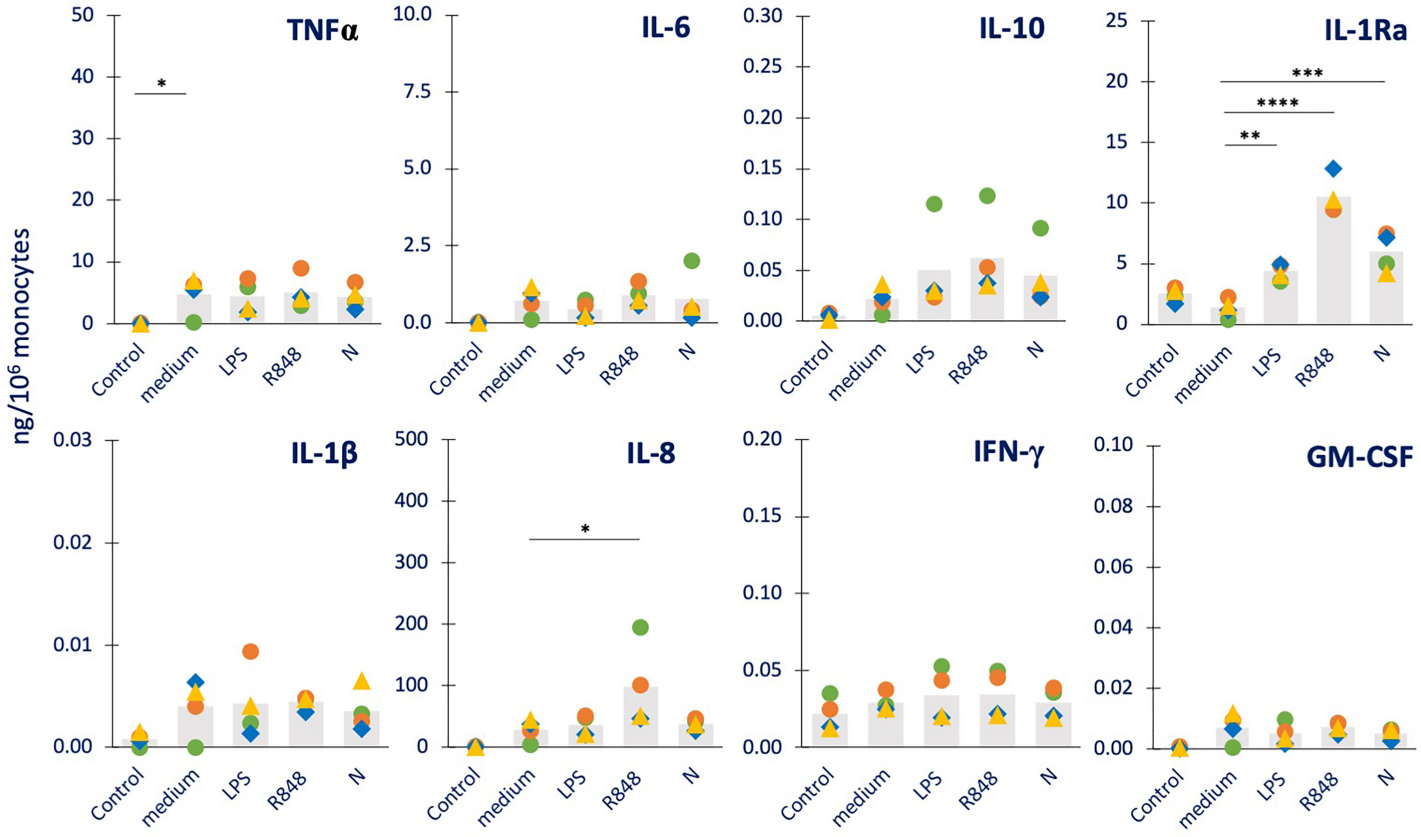
Innate immune memory response to a challenge with the N protein in human monocytes primed with bacterial or viral agents. Human monocytes isolated from blood of four individual donors (green, red, blue, and yellow symbols) were cultured for 24 h in culture medium alone (column “medium”) or containing LPS (1 ng/mL, column “LPS”, control bacterial agent), R848 (0.5 µg/mL, column “R848”, control virus-like agent) or N (1 µg/mL, column “N”). Cells were then washed and rested for 7 days in the absence of stimuli, then challenged for 24 h in fresh medium alone or containing 5 µg/mL N. The production of TNFα, IL-6, IL-10, IL-1Ra (upper panel from right to left) and IL-1β, IL-8, IFN-γ, GM-CSF (lower panel from right to left) was measured in the 24 h supernatants by ELISA. The values from cells that received no challenge are included in each panel as “Control” and encompass the values obtained from primed and unprimed cells (which did not differ, confirming the return to baseline after the resting period). Data are presented as individual donors’ values (colored symbols) and as mean of the individual values (gray columns). Statistical significance: * *p <*0.05; ** *p <*0.01; *** *p* < 0.001; **** *p <*0.0001.

Eventually, we investigated the possibility that exposure to N could induce a memory response in previously primed cells. Thus, we have measured the production of cytokines in response to N (5 µg/mL) in monocytes previously primed with medium alone or containing LPS (1 ng/mL), R848 (0.5 µg/mL) or N (1 µg/mL). The data in **Figure 4** show that challenge with N could induce a significant production of TNFα, and measurable levels of IL-6, IL-10, IL-1β, IL-8 and GM-CSF in unprimed cells (of 3/4 donors), while unable to increase the constitutive production of IL-1Ra and IFN-γ (columns “medium”). None of the priming agents used (LPS, R848, N) was able to induce a memory that substantially changed the secondary response (although with strong inter-individual variability), except in the case of IL-1Ra, whose production in response to challenge with N was significantly increased in cells primed with LPS, R848 or N.

## Discussion and Conclusions

This study provides initial evidence that the major SARS-CoV-2 structural nucleocapsid (N) protein has the ability to induce an innate memory that changes the monocyte response profile upon re-challenge. It should be noted that these results are preliminary, since only cytokine production was examined, and their interpretation is likewise limited, since only four non-vaccinated donors are included.

Several studies have pointed to a possible role of innate memory, induced by live attenuated vaccines such as BCG, for preventing the severe effects of SARS-CoV-2 infection (29, 30). On the other hand, the innate immune/memory profile of monocytes/macrophages from convalescent or vaccinated subjects revealed that both the whole infective virus and the Spike protein encoding vaccine are able to induce a transcriptional and epigenetic reprogramming suggestive of establishment of innate memory (19, 23, 24). Notably, *in vitro* challenge of blood leukocytes from convalescent individuals showed an increased production of the inflammatory/activating cytokines IL-1β and IL-6, based on an extensive epigenetic reprogramming of both CD14+ and CD16+ monocytes (24). Indeed, inhibitors of IL-6 (e.g., the anti-IL-6 receptor Tocilizumab) and of IL-1β (Anakinra) have been used in the therapy of COVID-19. While the treatment with Tocilizumab showed contradictory results regarding its efficacy (31–33), the use of the recombinant form of the IL-1 receptor antagonist IL-1Ra Anakinra showed encouraging results in decreasing clinical parameters and reducing overall mortality (34–36).

Bearing in mind that these results are preliminary, the major finding of our study is that one of the SARS-CoV-2 proteins, the nucleocapsid protein N, can induce innate memory in human monocytes (from subjects that were not previously infected by SARS-CoV-2 or vaccinated), and that this memory was almost exclusively represented by an increased capacity to produce the IL-1 inhibitor IL-1Ra. While confirming that innate memory is non-specific (the same memory response in terms of increased IL-1Ra production is triggered by challenge with LPS, R848 or N), this finding points to the importance of the N protein in stimulating an anti-inflammatory compensatory mechanism to control the cytokine storm and the tissue inflammation caused by the infection. Only in response to a strong viral-like challenge (R848), priming with N could also result in enhanced production of IL-1β. This suggests that the memory induced by N is preferentially active in anti-viral responses and includes the potentiation of a very important defensive effector molecule while, at the same time, being able to control unwanted inflammation through the enhanced production of anti-inflammatory IL-1Ra. Upregulation of IL-1Ra is expected to inhibit IL-1-dependent inflammation but also the entire inflammatory cascade initiated by IL-1β (37, 38). Conversely, a tendency of decreased production of the inflammatory cytokines TNFα and IL-6 was observed in cells primed with the two spike protein subunits S1 and S2 and challenged with the R848. Since only two donors could be examined for S1 and S2 priming, this tendency cannot be considered reliable. However, it may suggest that different parts of the virus can prime the innate immune system towards a milder secondary reaction by using different mechanisms (increase of anti-inflammatory reactions induced by N protein priming and a concomitant decrease of inflammatory responses induced by Spike protein priming). The fact that the whole virus could not induce any memory response may be explained by the cross-regulating effects of the different viral components or by changes induced by the inactivation process.

**Table 1** summarizes the findings reported in this study and highlight the strong and consistent potentiation effect of priming with the N protein on IL-1Ra production, which may underlie a less severe response to secondary infections, with a better control of innate/inflammatory effector mechanisms.

**Table 1:** Summary of the capacity of SARS-CoV-2 and its proteins to induce innate memory in human monocytes.

Priming and challenge/memory stimuli		Cytokine production*							
		TNFα	IL-6	IL-10	IL-1Ra	IL-1β	IL-8	IFN-γ	GM-CSF
*Priming with*	*Memory response to*								
SARS-CoV-2	LPS (bacteria)	no	no	no	no	no	no	no	no
	R848 (viruses)	no	no	no	no	no	no	no	no
N	LPS (bacteria)	no	no	no	↑↑	no	no	no	no
	R848 (viruses)	(↑)	no	no	↑↑	↑	no	no	(↑)
	N (SARS-CoV-2)	no	no	no	↑↑	no	no	no	no
S1	LPS (bacteria)	(↑)	no	no	no	no	no	no	no
	R848 (viruses)	(↓)	(↓)	no	no	no	no	no	(↓)
S2	LPS (bacteria)	no	no	no	no	no	no	no	no
	R848 (viruses)	(↓)	(↓)	no	no	no	no	no	(↓)
R848	R848 (viruses)	↑	↑	no	↑↑	↑	no	↑	↑
	N (SARS-CoV-2)	no	no	no	↑↑	no	↑	no	no

*Cytokine production is expressed as increase (↑ or ↑↑), indicating increase and strong increase, respectively), decrease (↓) or no change (no) compared to medium-exposed cells. Symbols within parentheses indicate dubious results, i.e., those in which the trend was observed in 3/4 donors (for N priming) and in which only two donors could be examined (for S1 and S2 priming).

## Data Availability Statement

The original contributions presented in the study are included in the article/**Supplementary Material**. Further inquiries can be directed to the corresponding author.

## Ethics Statement

Institutional Review Board Statement: The study was conducted according to the guidelines of the Declaration of Helsinki. The protocol was approved by the Regional Ethics Committee for Clinical Experimentation of the Tuscany Region (Ethics Committee Register n. 14,914 of May 16, 2019). Informed Consent Statement: Informed consent was obtained from all subjects involved in the study.

## Author Contributions

PU, SG, DB and PI planned the work. PU and SG performed the experiments and prepared the figures. PI and DB supervised the study. PI performed statistical analysis. DB critically assessed the results; all authors contributed to writing the manuscript and have read and agreed to the published version of the manuscript. All authors contributed to the article and approved the submitted version.

## Funding

This research work was supported by the European Commission’s Joint Research Centre (JRC) within the Consumer Products Safety of the Directorate of Health, Consumers and Reference Materials and partly funded the Horizon 2020 Framework Programme under the project ATAC-H2020-SC1-PHE-CORONAVIRUS-2020 (GA 101003650). Additional support was provided by the President’s International Fellowship Programme (PIFI) of CAS (to DB) and by the Italian project MIUR/PRIN-20173ZECCM (to PI).

## Conflict of Interest

The authors declare that the research was conducted in the absence of any commercial or financial relationships that could be construed as a potential conflict of interest.

## Publisher’s Note

All claims expressed in this article are solely those of the authors and do not necessarily represent those of their affiliated organizations, or those of the publisher, the editors and the reviewers. Any product that may be evaluated in this article, or claim that may be made by its manufacturer, is not guaranteed or endorsed by the publisher.
