# Supplementary material for: The SARS-CoV-2 Nucleoprotein Induces Innate Memory in Human Monocytes
Source: Front Immunol. 2022 Jul 19;13:963627. doi: 10.3389/fimmu.2022.963627 (PMC9343583; doi:10.3389/fimmu.2022.963627)
Supplement: Supplementary file 1 [file Image_1.pdf]

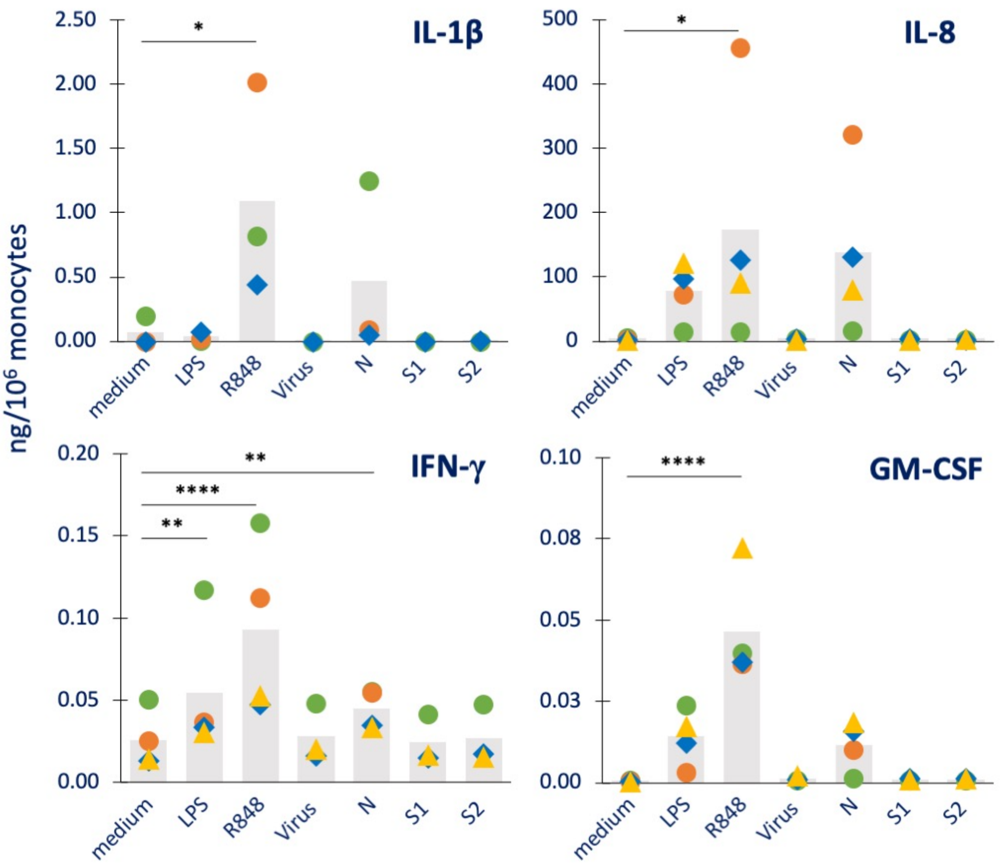

Supplementary Figure S1. Primary innate immune response to inactivated SARS-CoV-2 or its proteins in human monocytes.

Human monocytes isolated from blood of four individual donors (green, red, blue, and yellow symbols) were cultured for 24 h in culture medium alone or containing the inactivated SARS-CoV-2 virus (5 x10^5 copies), or the viral proteins N, S1, S2 (all at 1 µg/mL). The production of IL-1β (upper left), IL-8 (upper right), IFN-γ (lower left) and GM-CSF (lower right) was measured in the 24 h supernatants by ELISA. Medium alone was used as baseline value, LPS (1 ng/mL) and R848 (0.5 µg/mL) were used as positive controls. Data are presented as individual donors' values (colored symbols) and as mean of the individual values (gray columns). Statistical significance: * p <0.05; ** p <0.01; **** p <0.0001.
