# Supplementary material for: The SARS-CoV-2 Nucleoprotein Induces Innate Memory in Human Monocytes
Source: Front Immunol. 2022 Jul 19;13:963627. doi: 10.3389/fimmu.2022.963627 (PMC9343583; doi:10.3389/fimmu.2022.963627)
Supplement: Supplementary file 2 [file Image_2.pdf]

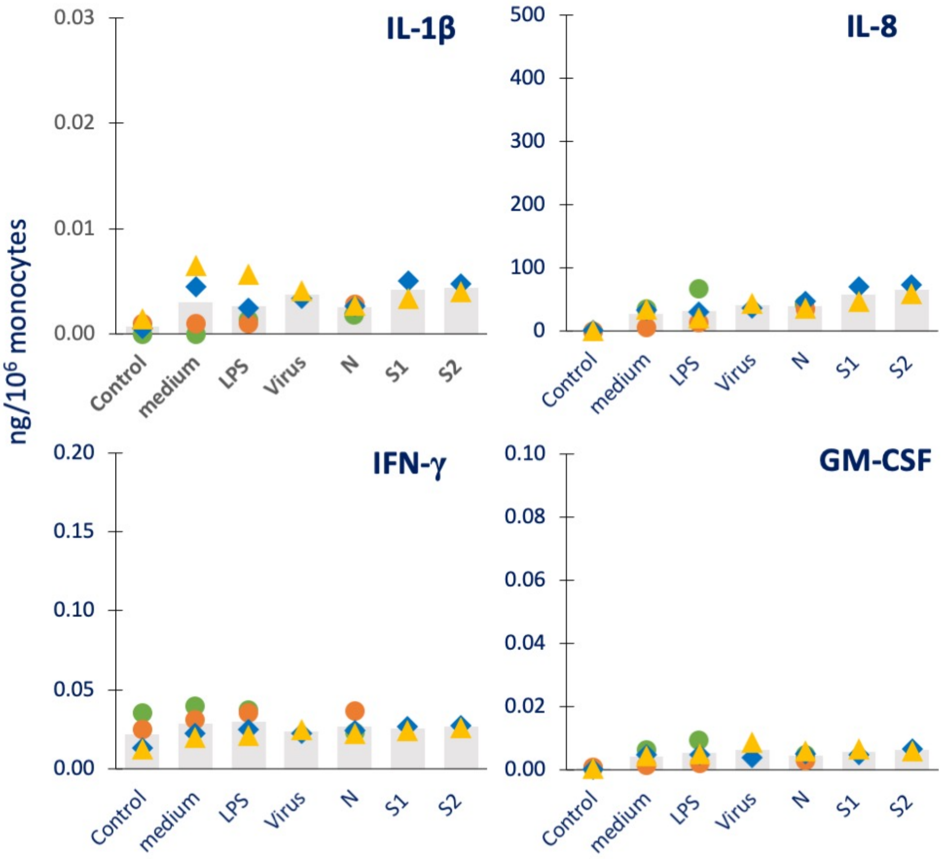

Supplementary Figure S2. Innate immune memory response to a bacterial challenge in human monocytes primed with inactivated SARS-CoV-2 or its proteins.

Human monocytes isolated from blood of four individual donors (green, red, blue, and yellow symbols) were cultured for 24 h in culture medium alone or containing LPS (1 ng/mL, positive bacterial control), the inactivated SARS-CoV-2 virus (5 x10^5 copies), N, S1, or S2 (all at 1 µg/ml). Cells were then washed and rested for 7 days in the absence of stimuli, then challenged for 24 h in fresh medium alone or containing 5 ng/mL LPS. The production of IL-1β (upper left), IL-8 (upper right), IFN-γ (lower left) and GM-CSF (lower right) was measured in the 24 h supernatants by ELISA. The values from cells that received no challenge are included in each panel as "control" and encompass the values obtained from primed and unprimed cells (which did not differ, confirming the return to baseline after the resting period). Data are presented as individual donors' values (colored symbols) and as mean of the individual values (gray columns).
